# Bringing Home the Trash: Do Colony-Based Differences in Foraging Distribution Lead to Increased Plastic Ingestion in Laysan Albatrosses?

**DOI:** 10.1371/journal.pone.0007623

**Published:** 2009-10-28

**Authors:** Lindsay C. Young, Cynthia Vanderlip, David C. Duffy, Vsevolod Afanasyev, Scott A. Shaffer

**Affiliations:** 1 Department of Zoology, Ecology, Evolution and Conservation Biology Program, University of Hawaii, Honolulu, Hawaii, United States of America; 2 Hawaii Department of Land and Natural Resources, Division of Forestry and Wildlife, Honolulu, Hawaii, United States of America; 3 Department of Botany, Ecology, Evolution and Conservation Biology Program, University of Hawaii, Honolulu, Hawaii, United States of America; 4 British Antarctic Survey, Natural Environment Research Council, Cambridge, United Kingdom; 5 Institute of Marine Sciences and Dept of Ecology & Evolutionary Biology, University of California Santa Cruz, Santa Cruz, California, United States of America; 6 Department of Biological Sciences, San Jose State University, San Jose, California, United States of America; Institut Pluridisciplinaire Hubert Curien, France

## Abstract

When searching for prey, animals should maximize energetic gain, while minimizing energy expenditure by altering their movements relative to prey availability. However, with increasing amounts of marine debris, what once may have been ‘optimal’ foraging strategies for top marine predators, are leading to sub-optimal diets comprised in large part of plastic. Indeed, the highly vagile Laysan albatross (*Phoebastria immutabilis*) which forages throughout the North Pacific, are well known for their tendency to ingest plastic. Here we examine whether Laysan albatrosses nesting on Kure Atoll and Oahu Island, 2,150 km apart, experience different levels of plastic ingestion. Twenty two geolocators were deployed on breeding adults for up to two years. Regurgitated boluses of undigestable material were also collected from chicks at each site to compare the amount of plastic vs. natural foods. Chicks from Kure Atoll were fed almost ten times the amount of plastic compared to chicks from Oahu despite boluses from both colonies having similar amounts of natural food. Tracking data indicated that adults from either colony did not have core overlapping distributions during the early half of the breeding period and that adults from Kure had a greater overlap with the putative range of the Western Garbage Patch corroborating our observation of higher plastic loads at this colony. At-sea distributions also varied throughout the year suggesting that Laysan albatrosses either adjusted their foraging behavior according to constraints on time away from the nest or to variation in resources. However, in the non-breeding season, distributional overlap was greater indicating that the energy required to reach the foraging grounds was less important than the total energy available. These results demonstrate how a marine predator that is not dispersal limited alters its foraging strategy throughout the reproductive cycle to maximize energetic gain and how this has led to differences in plastic ingestion.

## Introduction

When searching for prey, an organism is expected to maximize its energetic gain, while minimizing energy expenditure. To accomplish this, organisms should increase the probability of detecting food patches by altering their movement path relative to the availability of prey [1], [2]. Unlike terrestrial environments, where physical barriers and geography often put constraints on movement, most oceanic environments are less obstructed. As a result, the movements of marine organisms that have high dispersal capability can potentially reflect their assessment of resource distribution and variation.

In the case of pelagic seabirds, like albatrosses and petrels, that nest on remote oceanic islands [3] one would predict that breeding populations on distant islands should maximize energetic gain by exploiting available resources closest to their respective breeding colony [4]. Consequently, regional differences in diet associated with the differences in foraging ranges of a particular population may be observed due to difference in prey distribution. Upon completion of breeding, birds are no longer tied to a nest and so greater population mixing is likely, even for populations separated by several thousand kilometers. Thus, movement patterns during this phase of the life cycle would perhaps be more determined by variations in prey distribution [5], [6], [7].

The at-sea foraging patterns of the highly mobile Laysan albatross (*Phoebastria immutabilis*) have been well documented for breeding individuals [8]–[13]. Their annual reproductive cycle is marked by several phases: during incubation and early chick rearing stages Laysan albatrosses are central-place foragers, but later, when they are less constrained by nest visitations, Laysan albatrosses forage throughout the North Pacific [8], [11]–[13]. However, it is unknown whether all breeding colonies in the North Pacific exploit the same or similar resources or habitats year round, even though they may be well within their dispersal potential, or if colonies utilize ‘locally’ available resources since previous studies have focused on a single breeding colony [8], [13].

Central place foraging, coupled with huge aggregations of birds at the breeding colonies, likely exerts predation pressure on local prey stocks, and potentially amplifies intra-specific competition at the colonies. This is thought to possibly lead to localized prey depletion around breeding colonies which has been called ‘Ashmoles Halo’ [14]. In order to maximize food availability and minimize intra-specific competition, albatross colonies should be spaced so that the foraging zones of the birds do not greatly overlap. However, in the Hawaiian islands where 99% of Laysan albatross populations breed [10], colonies are not evenly distributed and the inter-colony distances are often much smaller than their maximum foraging range [15]. If prey resources throughout the North Pacific Basin are evenly distributed, then colony-specific foraging radii will overlap widely because the distance between colonies is shorter than the birds foraging radius [8]. However, a more likely scenario given the heterogeneity of the North Pacific, is that Laysan albatrosses should exploit suitable resources closest to their respective colony during the breeding period, even though they are capable of travelling to virtually any foraging ground in the North Pacific.

**Figure 1:**
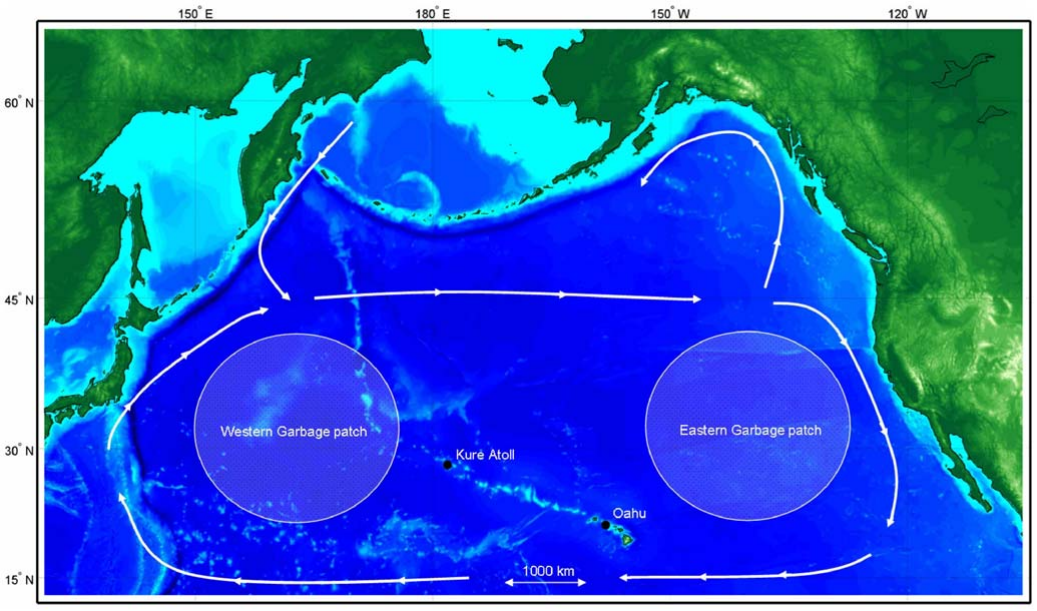
Study sites relative to major current systems and ‘garbage patches’ in the North Pacific Ocean. Arrows indicate direction of currents and shaded areas denote the locations of the putative garbage patches.

**Figure 2:**
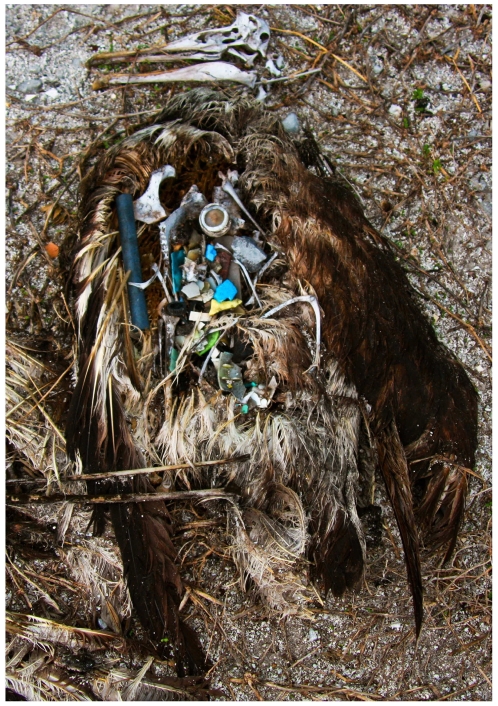
Photograph of a dead Laysan albatross chick with plastic in its stomach.

The variable diet and flexible foraging strategy of Laysan albatrosses (utilizing both scavenging and live capture; [10], [16]) indicate that this species is a generalist. Ironically, the flexible foraging strategy of this species has potentially led to a decrease in their foraging efficiency as they commonly ingest large amounts of plastic, which is in turn fed to their chicks [17]–[20]. However, it is unclear how often Laysan albatrosses encounter or ingest plastic, whether plastic is mistaken for prey, has natural food attached to it, or is consumed to assist in digestion as is sometimes done with pumice [17]–[20]. Unfortunately, plastic ingestion leads to mechanical blockage of the digestive tract, reduced food consumption, satiation of hunger, and potential exposure to toxic compounds. While there have been documented detrimental effects on the growth rates and fledging masses of chicks, it is still unclear what levels of mortality are caused by plastic ingestion [17]–[20]. What is clear is the source of the plastic: there is now so much floating marine debris accumulated in the North Pacific gyre, that is it known as the ‘great garbage patch’ [21], [22], [23]. This patch consists of high densities of floating plastic debris, particularly between 20°–40° N, within a few hundred kilometres of the coast and in the gyre centres, between the tropical and subarctic waters. This area of concentrated debris consists of two accumulations: the ‘Western Garbage Patch’ that occurs off Japan and ‘Eastern Garbage Patch’ residing between Hawaii and California (http://coastwatch.pfel.noaa.gov) [24], [25] that correspond to the locations of two sub-gyres within the North Pacific Gyre [26], connected by a narrower band of marine debris north of the Hawaiian archipelago (Figure 1). Much has been written in the popular press about plastic ingestion by Laysan albatrosses (Figure 2), however, relatively few empirical studies have examined this phenomenon and whether it is species wide, or if this is confined to certain populations.

Here we examine whether Laysan albatrosses nesting on widely separated islands exploit resources closer to their breeding colonies during the breeding season, how their foraging locations change throughout the reproductive cycle, and whether this leads to differences in plastic ingestion represented in their boluses. In addition to the potential of seabird boluses to monitor plastic in our oceans, determining how and where marine organisms come into contact with marine debris could have implications for the design of management strategies that mitigate its environmental impact. To do this, we combined the use of electronic data logging devices to determine at-sea distributions with the collection of chick boluses to evaluate the differences plastic ingestion.

## Results

### Geolocator recovery rates and effects on reproductive success

Fourteen and 32 tags were recovered from Oahu and Kure Atoll respectively, which resulted in an overall tag recovery rate of 54% (*N* = 46/85). In most cases, geolocators that were not recovered had broken free of the leg band as a result of cable tie failure, despite the bird itself returning. Of the 46 tags recovered, only 22 produced data even after failed batteries were recovered (48%; 10 from Oahu, 11 from Kure). While the cause of the electronic failure of the geolocators is unknown, it is possible that saltwater intrusion occurred as a result of epoxy expansion at higher temperatures which allowed water to leak onto the electrical components. Between tag loss and electrical failure, the proportion of loggers that produced data was 26% (22/85).

Deployment of geolocators had no detectable short-term effect on the birds. Tagged Laysan albatrosses on Oahu had similar reproductive success (71%, *N* = 10/14 nests were successful), as well as resight probabilities the following year (86%, *N* = 24/28 individuals) compared to non-tagged birds (reproductive success = 55%, *N* = 12/22, X^2^ = 1.03, df = 1, *p* = 0.311; resight probability  = 86%, *N* = 30/35, X^2^ = 0.00, df = 1, *p* = 1.00). Multiple birds on Kure Atoll developed minor calluses and chafing on the leg as a result of unsecured cable ties rotating under the leg band. These were temporarily removed immediately after deployment, modified to prevent movement of the cable tie, and re-deployed, which appeared to eliminate further chafing.

### Spatial associations of each colony

**Figure 3:**
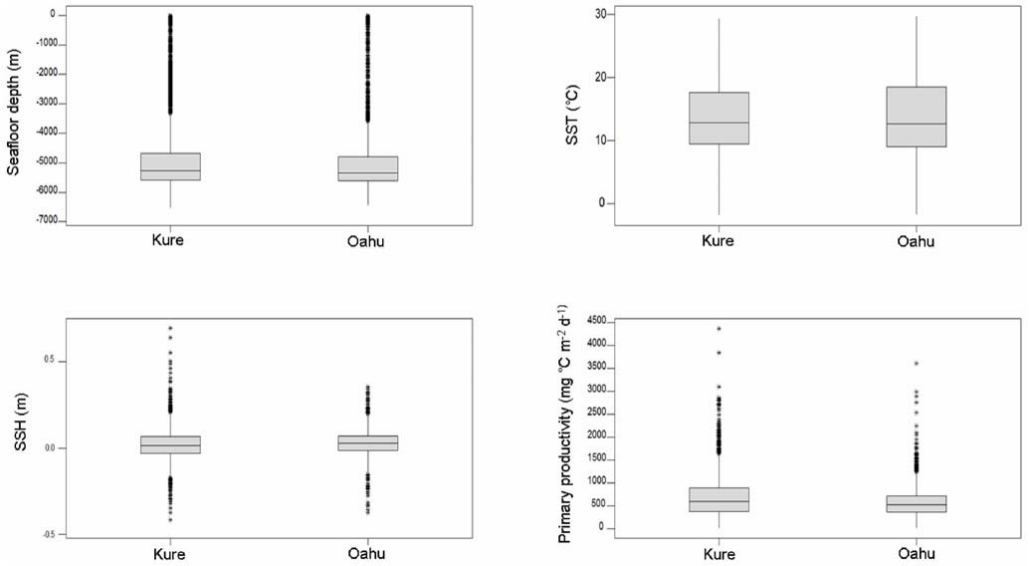
Mean oceanographic parameter values for foraging Laysan albatrosses from Kure and Oahu. Boxplots of bathymetry, SST, SSH, and primary productivity of oceanographic habitats visiting by birds tracked from each colony.

**Figure 4:**
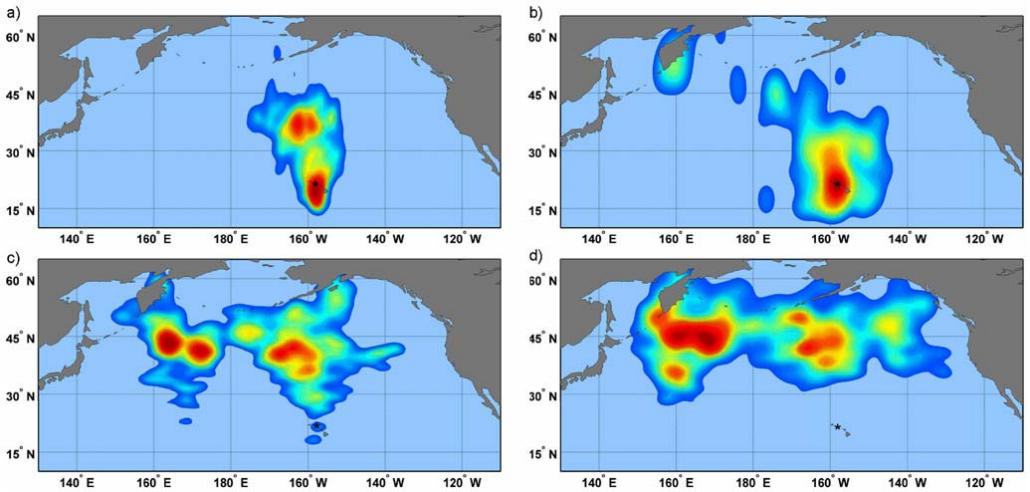
At-sea utilization distribution kernels for Laysan albatross foraging from Oahu. Kernels increase in 2% increments from 2%–95% with increasingly warmer tones represent the highest utilization distribution (2%). Kernels are shown for the a) incubation b) chick guard c) post-guard and d) non-breeding stages. Colony location is indicated by an asterisk.

**Figure 5:**
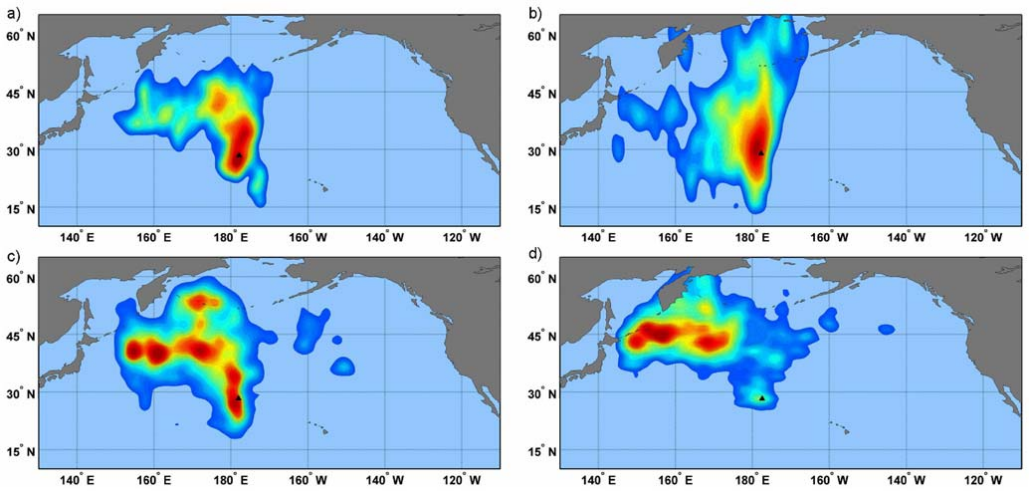
At-sea utilization distribution kernels for Laysan albatross foraging from Kure Atoll. Kernels increase in 2% increments from 2%–95% with increasingly warmer tones represent the highest utilization distribution (2%). Kernels are shown for the a) incubation b) chick guard c) post-guard and d) non-breeding stages. Colony location is indicated by a triangle.

**Figure 6:**
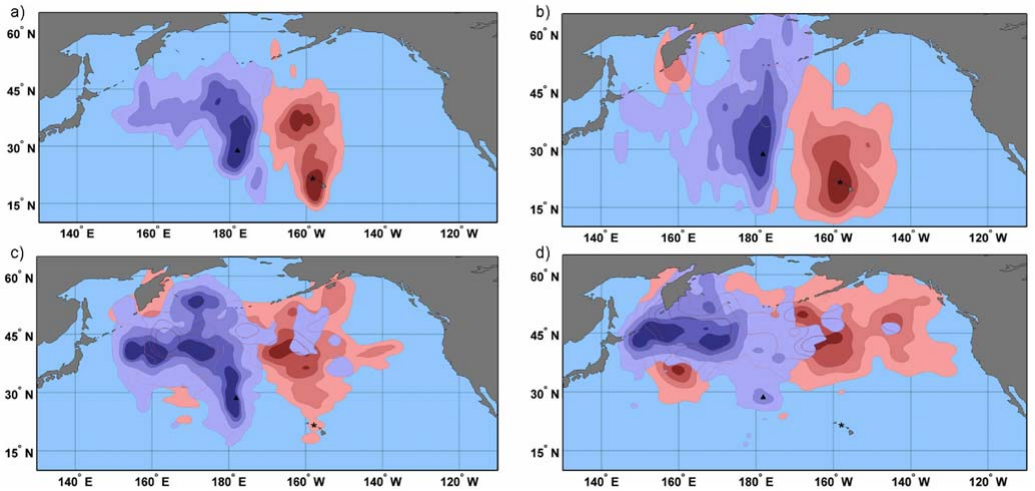
Overlap of kernel density estimates for Laysan albatross foraging from Kure and Oahu during phases of the reproductive cycle. Red tones represent Kure Atoll, blue represent Oahu and contour lines represent the 95%, 75%, 50%, and 25% kernel estimates during the a) incubation b) chick guard c) post-guard and d) non-breeding stages. Kure colony location is indicated by a triangle, and Oahu colony location is indicated by an asterisk.

Laysan albatrosses from both colonies had similar associations with productivity regimes and depth domains quantified from bathymetric features, sea surface temperature (SST), sea surface height (SSH) and primary productivity (Figure 3). During incubation and early chick rearing, birds from both colonies foraged in pelagic (depth: >4000 m), oligotrophic (primary productivity: <600 mg C m^−2^ d^−1^) tropical to sub-tropical waters (SST: <16°C). During the post-guard stage of chick rearing, birds from both colonies began venturing into cooler, more productive waters further north of their respective colonies. Despite the similarities in the oceanographic features that birds from both colonies were exploiting, significant differences were found in the actual locations of foraging relative to their breeding colonies (Figures 3, 4, 5, 6, Table 1).

**Table 1:** Overlap of the core (25% and 50% kernel density estimates) ranges of Kure and Oahu Laysan albatrosses with each other, and with each garbage patch during the incubation, chick guard, post-chick guard and non-breeding periods.

Colony	Region	Incubation- UD 25%, 50%	Chick guard UD 25%, 50%	Post-chick guard UD 25%, 50%	Post-breeding UD 25%, 50%
Oahu	Kure	0, 0	0, 0	23, 28	38, 51
Oahu	E Pac	0, 0	0, 0	0.04, 2.5	4.0, 10.0
Oahu	W Pac	0, 0	0, 0	0, 6.9	9.6, 16.6
Kure	E Pac	0, 0	0, 0	0, 0	0, 0
Kure	W Pac	0, 0	0, 0	18.0, 15.0	0, 1.0

Overlap metrics range from 0 to 100%.

At the population level, albatross distributions during incubation and early chick rearing did not overlap in the core usage areas (50% and 25% kernels, Table 1, Figure 6). Birds from Oahu foraged north of the colony in the area of the North Pacific Transition Zone chlorophyll front [NPTZ, Figure 4; 27], but most did not pass within the area of the Eastern Garbage Patch. As the breeding season progressed, birds began venturing into the transition domain and subarctic waters, occasionally reaching the Aleutian Island chain. In contrast, albatrosses from Kure foraged north/northwest of the colony over the Emperor Seamounts, the area bounded by the Kuroshio and Oyashio current systems and occasionally over the Western Garbage Patch. As the breeding season progressed, birds moved farther north and west, foraging more in the subarctic frontal zone and the transition domain.

Conversely, during the post-chick guard stage when adults can spend increasing lengths of time away from the nest, or have failed in their nesting attempt, the core areas of Kure and Oahu birds began to overlap, primarily as a result of Oahu birds moving further west and the distributions of both colonies moving farther north. During the non-breeding season, from 38–50% overlap between the two colonies was observed in the 25% and 50% kernel density estimates. While both colonies expanded their foraging ranges throughout the season, Oahu birds appeared to have a bi-modal distribution in the non-breeding season with two distinct foraging areas at parallel locations on either side of the International Date Line (Figure 4), whereas Kure birds had a single core foraging area significantly west of the Date Line (Figure 5).

### Difference in plastic loads

**Figure 7:**
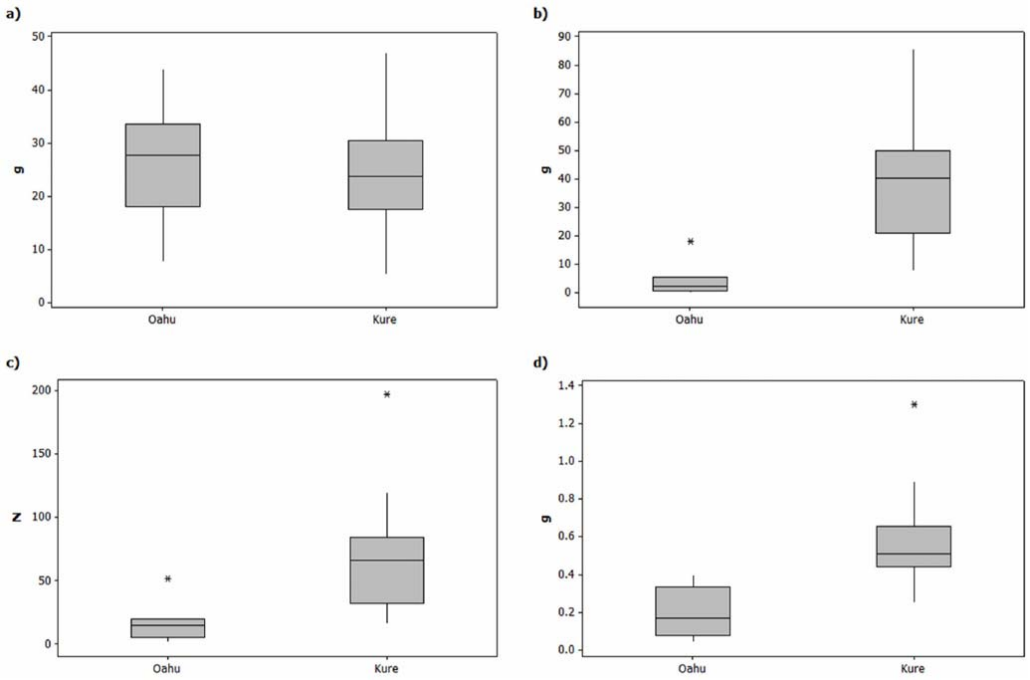
Comparison of plastic content in boluses from Laysan albatross chicks on Kure Atoll and Oahu. Boxplots of a) natural food mass, b) plastic mass, c) # plastic pieces and d) average plastic piece mass from Laysan albatross boluses on Kure and Oahu.

Every bolus examined in this study contained plastic. The amount of natural food found in the boluses of chicks on Kure and Oahu was similar in volume (30.75±3.72 ml vs. 32.18±5.63 ml; *p*>0.628) and mass (23.98±3.00 g vs. 26.14±3.94 g; *p>*0.745). However, the amount of plastic in Kure boluses was up to ten times higher than the amount of plastic in Oahu boluses (Figure 7): volume (53.67±6.38 ml vs. 5.26±2.50 ml; *p* = 0.0001), mass (38.03±5.32 g vs. 4.37±2.10 g; *p* = 0.0001), number of plastic pieces (70.6±11.5 vs. 17.4±5.5; *p* = 0.0004) and the average mass of plastic pieces (0.58±0.065 g vs. 0.20±0.047 g; *p* = 0.0001).

## Discussion

Distant colonies of Laysan albatrosses utilized similar oceanographic productivity regimes in widely separated areas, suggesting that this vagile animal is able to assess resources on large scales. For both colonies, similar associations with productivity and depth domains indicate that oceanographic characteristics of suitable foraging areas are part of an albatross's search image when they are trying to locate prey [8], [11], [13]. However, it is their ability to identify suitable areas closest to the colony when they are breeding which suggests that their assessment of resource variation also includes an assessment of the tradeoff between self-maintenance (longer trips) and chick provisioning (shorter trips) [8], and thus leads to foraging segregation during the early part of the reproductive cycle when they are required to return to the breeding colony frequently [5], [28].

During the non-breeding phase when Laysan albatrosses are not constrained by the time and energy required for breeding, there is a larger degree of overlap in foraging distributions between distant populations. This suggests that the energy required to reach the foraging grounds becomes less important than the total energy available on the foraging grounds themselves. Another possible explanation is that the colony on Oahu, which is a ‘new colony’ comprised almost entirely of immigrants from other colonies [29], [30] has immigrants from Kure Atoll, and that the bi-modal distribution in the non-breeding season may actually reflect Kure-born birds returning to their ancestral non-breeding foraging grounds. In either case, the results indicate that Laysan albatrosses are able to assess resource variation and alter their foraging strategies accordingly throughout their reproductive cycle as has been shown for other species [5], [6], [7], [31], [32], [33].

The fact that Laysan albatrosses from both colonies ingest plastic, unfortunately, suggests that the core areas where albatrosses prefer to forage contain substantial amounts of floating debris that are consumed. Previous studies have shown that the highest concentration of marine debris occurs in the spring and early summer months when the NPTZ moves south due to shifts in wind and weather changes [21]. This corresponds to the chick rearing season in Laysan albatross [10]. The finding that birds breeding on Kure Atoll fed their chicks, on average, ten times more plastic than birds breeding on Oahu suggests that putative Western Garbage Patch where the majority of Kure birds foraged may in fact be a just as much of a threat to marine life as the frequently discussed Eastern Garbage Patch. Furthermore, every bolus examined from Kure Atoll contained multiple pieces of fishing paraphernalia, while only two boluses on Oahu contained any evidence of fishing line or tools (despite recreational fishing adjacent to the breeding colony on Oahu), suggesting that the threat from fisheries not only comes from bycatch for this species but also from the consumption of fishing gear. It is unclear whether the Western Garbage Patch contains more trash than the Eastern Garbage Patch, or if the size and composition of the pieces are easier for the birds to ingest compared to those found in the Eastern Garbage Patch in addition to the finding that albatrosses from Kure spend a greater proportion of time foraging in this area.

Previous studies on seabirds have shown that comparisons of plastic accumulations within a species should have consistent biases when different populations are relatively synchronous in their breeding cycle, which Laysan albatross are. Studies on Northern Fulmars (*Fulmarus glacialis*) found that regional differences in foraging/nesting locations led to differences in plastic ingestion [34], [35]. While the most likely explanation of why the plastic loads differ between the two colonies examined in this study is due to seasonal differences in at sea distribution, it is possible that competition may be playing a role. There are approximately 75 Laysan albatross pairs on Oahu which may experience less intraspecific competition for prey than the 3,900 pairs breeding on Kure Atoll. The slightly smaller foraging area of Oahu-nesting birds during early chick-rearing stages might be an evidence for this. Since the amount of natural food in the boluses of both colonies is the same, adults on Kure Atoll may cope with this pressure by being less selective and thus misidentify more plastics for food. Further research identifying potential rates intraspecific competition between individuals in these two colonies will help to resolve this question. Additional research to determine the size, location(s) and contents of these floating debris patch(es) in the North Pacific would also greatly enhance our ability to determine their impacts on marine life.

Environmental heterogeneity in marine, as well as terrestrial systems, affects animal movements on a range of scales [5], [6], [7], [31], [32], [33], [36]. Our results demonstrate that Laysan albatrosses are able to assess prey availability over large scales and make foraging decisions based on the energy required to reach feeding grounds throughout their breeding cycle. While the preferred foraging locations during the early breeding season are different for each population, these predators appear to seek out common ecological characteristics, leading to the use of similar habitats by albatrosses from the two colonies in the non-breeding season when they are not constrained by breeding. Studying movements at a smaller scale in relation to resource distribution, and at multiple colonies over multiple years will be crucial to fully understand scale-dependent adjustments and the ultimate foraging distribution of these animals. Future studies of foraging behavior would also benefit from not only monitoring plastics ingested, but also quantifying natural diet to ascertain regional differences. The large range and potentially long-term retention of ingested plastic indicate that albatross may prove to be a useful species for sampling marine debris and other pollutants in the North Pacific Ocean [35], and as such, efforts should be made to continue monitoring their plastic ingestion and foraging patterns.

## Materials and Methods

### Data logger deployments

Global Location Sensing data loggers (or geolocators) are small microprocessor-based devices that determine a geographic position on the globe from the establishment of local noon or midnight to estimate longitude and day length at the estimated longitude to derive latitude [37], [38]. These devices measures light every minute, and record the maximum light level at the end of every 10 minute period. These periods are then compressed and filtered to produced two location fixes per day.

**Figure 8:**
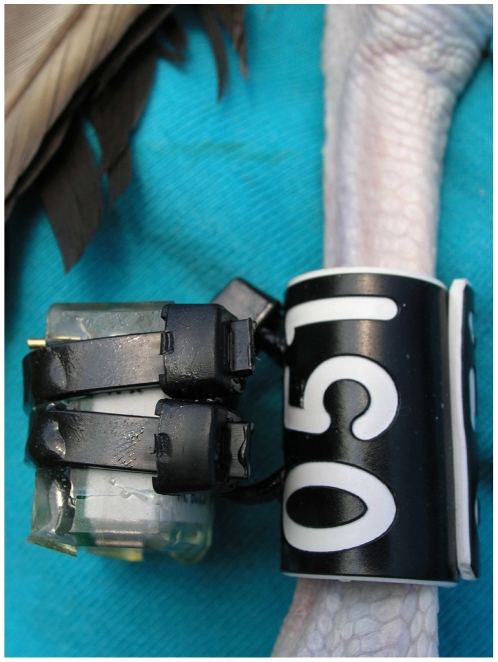
Example of geolocator attachment methods on a Laysan albatross.

We deployed leg mounted geolocators on Laysan albatrosses breeding at two colonies: Oahu in the main Hawaiian Islands, and Kure Atoll 2,150 km away in the Northwestern Hawaiian Islands (Figure 8). Model MK3 geolocators (9 g) manufactured by the British Antarctic Survey (BAS) were attached to a plastic leg band by pre-drilling holes and threading cable ties through the leg band to secure the geolocator to it (Figure 8). The contact points were then sealed with marine grade epoxy and the leg band with the geolocator attached was placed around each bird's tarsus.

On Oahu, 14 pairs (N = 28) of incubating or brooding adult Laysan albatrosses at Kaena Point Natural Area Reserve (21° 34′N, 158°16′W) were outfitted with geolocators in February 2005 representing 39% (N = 14/36 nests) of breeding adults that year. The reproductive outcomes of all pairs were tracked from egg laying to fledging to monitor the effects of tagging on reproductive success. On Kure Atoll (28°23′N, 178°17′W), 28 pairs, plus one additional breeding adult (N = 57) were outfitted with geolocators in May 2005 during the late chick-rearing phase. Logistical limitations of working at a remote island like Kure Atoll prevented us from deploying the loggers at the same time of year and monitoring the reproductive fate of these birds.

### Geolocator filtering and spatial data analysis

Geolocators were retrieved at various time periods from January 2006- June 2007, depending on when tagged birds returned to the colony. Downloaded data were decompressed using BASTrak v12 software (BAS) and light curves (i.e. to establish sunrise and sunset and thus local noon or midnight) were analyzed using Transedit software (BAS) using a sunrise angle of −2.5° for Oahu, and −3° for Kure. Any locations produced from light curves with interruptions or interference around the times of sunset or sunrise (usually as a result of shading of the sensor) were noted during processing and excluded if obviously anomalous. Final position coordinates were generated by BirdTracker (BAS). Validation studies on albatrosses indicate that location error using this methodology range from 200±150 km from true location [9], [38].

Purpose-built routines created in Matlab R2008b (The MathWorks, Natick, Massachusetts) were used to filter spurious positions and to integrate remotely-sensed environmental data following established protocols [9], [35], [38]. Given the inaccuracy of latitude estimation during equinoxes [9], [37], [38], [39], location fixes on ten days of either side of the equinoxes were excluded from the analysis. Three subsequent filters were applied to data once the equinoxes had been removed: one to filter out unrealistic locations based on excessive travel rates (>500 km in 12 hours), a second to remove any remaining points occurring in locations where Laysan albatrosses have not been recorded previously (latitude and longitudes), and a third to remove any points that occurred over continental land masses [9], [36]–[39]. Of 14,281 locations produced, 72% (N = 10,314) were kept after filtering.

Estimates of bathymetry, and remotely-sensed sea surface temperature (SST), sea surface height (SSH), and primary productivity were calculated for each bird location based on previously described methods [36], [40]. These data allowed us to describe the frequency of occurrence that Laysan albatrosses spent within specific water masses, productivity regimes, and depth domains. To assess the degree of spatial overlap at sea of albatrosses from each colony, we derived kernel density distribution estimates [40] from unsmoothed (i.e. non-interpolated) locations using the Iknos toolbox (Y. Tremblay unpublished) developed in MatLab. This routine converted geographic coordinates to Cartesian coordinates using a Lambert Cylindrical Equal Area projection [41] and created 2-D kernels based on an 80 km grid cell size. The kernel smoothing parameter (h) was based on an adaptive method [42]. The density of albatrosses within a kernel was determined by dividing the number of observations by the number of individuals contributing to those observations (i.e. bird effort). We then quantified the spatial overlap of the 95% (foraging range), 75%, 50% (focal region), and 25% (core) kernel polygons representing the distribution of the albatrosses from each colony during each breeding stage and non-breeding period.

### Bolus collection and analysis

Boluses are the non-digestible parts of the albatross's diet (e.g. squid beaks, otoliths, plastic etc) that are regurgitated by chicks just prior to fledging. The boluses therefore represent a non-invasive method to evaluate the prey acquisition of parents as reflected by the diet of the chicks [10]. It is assumed that a single chick produces a single bolus and that the majority of plastics fed to chicks by adults were acquired during the breeding period. Naturally regurgitated boluses were collected from eight chicks on Oahu and 15 from Kure Atoll during the time interval when adults were tracked and from the area where chicks of tracked adults were found to attempt to sample the offspring of tracked adults. Due to the presence of rats in the colony on Oahu, only boluses that appeared to be completely intact were collected as rats have been observed to scavenge the natural food items in the bolus (L. Young pers. obs). The boluses collected represent 17% of the chicks on Oahu during the two year time period that adults were tracked (N = 8/48). The boluses collected on Kure represent approximately 0.4% (N = 15/3,900) of the population of breeding pairs [15].

Boluses were soaked for 24 hours in water and then sorted according to natural food items (such as flesh, squid beaks and lenses) and plastic items. Wet mass and displacement volume were measured, and plastic items were further categorized into the number and the average mass of pieces per bolus. Mass and volume of natural vs. plastic items in each bolus, as well as the number and average mass of the plastic pieces were compared between Oahu and Kure by using a Mann-Whitney U-test in Minitab 14.
